# Identification and RNAi-Based Functional Analysis of Four Chitin Deacetylase Genes in *Sogatella furcifera* (Hemiptera: Delphacidae)

**DOI:** 10.1093/jisesa/ieab051

**Published:** 2021-08-01

**Authors:** Xi-Bin Yang, Cao Zhou, Ming-Fu Gong, Hong Yang, Gui-Yun Long, Dao-Chao Jin

**Affiliations:** 1 Institute of Entomology, Guizhou University; Guizhou Provincial Key Laboratory for Agricultural Pest Management of Mountainous Regions, Guiyang, China; 2 Scientific Observing and Experimental Station of Crop Pests in Guiyang, Ministry of Agriculture and Rural Affairs of the People’s Republic of China, China; 3 College of Life Science, Chongqing Normal University, Chongqing, China; 4 College of Tobacco Science of Guizhou University, Guiyang, China

**Keywords:** *Sogatella furcifera*, chitin deacetylase, molting, wing expanse, chitin biosynthesis

## Abstract

Chitin deacetylases (CDAs) are chitin-degrading enzymes that play a key role in insect molting. In this study, we identified and characterized four full-length cDNAs of CDAs from *Sogatella furcifera* (Horváth). Developmental expression showed that *SfCDA1* and *SfCDA2* were expressed at all nymph developmental stages, *SfCDA3* and *SfCDA4* were mainly expressed in the third-instar to fifth-instar nymph stages, whereas tissue-specific analyses indicated that four CDA genes were mainly high expressed in the integument and head during the fifth-instar nymph. RNA interference (RNAi) results revealed that *SfCDA1, SfCDA2*, and *SfCDA4* are associated with molting defect and high mortality with nymph–adult molting. Furthermore, transcripts of chitin synthase 1 variants (*SfCHS1*, *SfCHS1a*, and *SfCHS1b*) were significantly downregulated and causing significant changes in the expression levels of trehalases (*TRE1* and *TRE2*) in the *SfCDA1*, *SfCDA2*, and *SfCDA4* dsRNA treatment groups. By contrast, no significant phenotypic characteristics were observed after ds*SfCDA3* injection. Taken together, our results suggest that *SfCDA1*, *SfCDA2*, and *SfCDA4* play a vital role in nymph-adult transition, and these genes could regulate chitin biosynthesis expression levels.

Chitin, a linear polymer of *N*-acetylglucosamine linked with *β*-1,4 glycosidic bonds, is widely distributed in fungi, nematodes, insects, brachiopods, and mollusks (Merzendorfer 2006). In insect, chitin has been a major component of the integument, peritrophic membrane (PM), and tracheal tisgsues, and it plays an important role in maintaining the shape and protecting against external forces and is critical for molting (Moussian 2010, Merzendorfer 2011). The periodic degradation and synthesis of chitin is essential for insects’ proper growth and development (Arakane and Muthukrishnan 2010, Zhu *et al.* 2016).

Chitin deacetylases (EC3.5.1.41, CDAs) are key enzymes involved in chitin metabolism, belonging to carbohydrate esterase family 4 (CE4). They catalyze the *N*-deacetylation of chitin to form chitosan (Tsigos *et al.* 2000). The first insect *CDAs* sequence was identified from the midgut of *Trichoplusia ni* (Lepidoptera: Noctuidae) (Guo *et al.* 2005). Since then, several CDAs have been identified in many insect orders, including Lepidoptera (Zhong *et al.* 2014, Tetreau *et al.* 2015), Coleoptera, Hymenoptera, Dipteran (Dixit *et al.* 2008), Hemiptera (Xi *et al.* 2014), and Orthoptera (Yu *et al.* 2016, Ding *et al.* 2014). Insect CDAs were divided into five groups (I–V) based on sequence homology and domain structures. Groups I and II include a chitin-binding domain (CBD), a low-density lipoprotein receptor domain (LDLa), and a deacetylase-like catalytic domain (CDA). Groups III and IV contain a CBD and a CDA, but group V only has a CDA (Tetreau *et al.* 2015). Group I CDAs (*CDA1* and *CDA2*) play a critical role in molting, as demonstrated by RNA interference (RNAi) knockdown in *Tribolium castaneum* (Coleoptera: Tenebrionidae) (Arakane *et al.* 2009), *Stegobium paniceum* (Coleoptera: Anobiidae) (Yang *et al.* 2018), *Choristoneura fumiferana* (Lepidoptera: Tortricidae) (Quan *et al.* 2013), *Nilaparvata lugens* (Hemiptera: Delphacidae) (Xi *et al.* 2014), and *Locusta migratoria* (Orthoptera: Acrididae) (Yu *et al.* 2016, Yu *et al.* 2018). In *Drosophila melanogaster* (Diptera: Drosophilidae), a serpentine (serp) mutation in *CDA1* or a vermiform (verm) mutation in *CDA2* leads to elongated and tortuous tracheal tubes (Luschnig *et al.* 2006, Wang *et al.* 2006). Group V CDAs, a midgut PM protein, and group V CDAs are involved in changes to the physicochemical properties of chitin in the midgut PM of *Mamestra configurata* (Lepidoptera: Noctuidae) and *Bombyx mori* (Lepidoptera: Bombycidae), which affects the binding degree and integrity of proteins (Toprak *et al.* 2008, Liu *et al.* 2019). Despite these numerous reports, the number and function of *CDA* genes in *Sogatella furcifera* (Horváth) remain unknown.

The white-backed planthopper (WBPH), *S. furcifera*, is an important pest of rice and causes severe losses in the rice production by sucking plant juices, oviposition, and transmitting viruses (Zhou *et al.* 2008). Currently, control of *S. furcifera* has mainly relied on insecticides, but due to its irrational usage, *S. furcifera* has developed resistance against many insecticides and caused agricultural environmental pollution (Zhang *et al.* 2017, Nakata *et al.* 2019). Therefore, it is urgent to explore new pest-control strategies. For example, RNAi technique is able to effectively knock down expression of vital genes insects and it holds considerable potential for controlling pest insects (Niu *et al.* 2018). Because chitin is absent in vertebrates, CDAs genes could be considered as targets for RNAi-based pest control.

In this study, we identified and characterized the full-length cDNA sequences of four *CDA* genes from *S. furcifera* and investigated their expression profiles at different developmental stages and tissues. Furthermore, RNAi of CDAs genes was performed to elucidate roles during nymph-adults molting.

## Materials and Methods

### Insects Rearing

*Sogatella furcifera* were collected in 2013 from a rice field in the Hua Xi district, Guiyang, Guizhou Province, China. All insects were reared on TN1 rice seedlings in a climate chamber at a temperature 25 ± 1°C and 70 ± 10% relative humidity (RH) with a photoperiod of 16 h (light) and 8 h (dark).

### Cloning and Sequencing

First, the *S*. *furcifera* genomic and transcriptomic annotation database (Wang *et al.*, 2017, Zhou *et al.*, 2018) was identiﬁed. Second, reported *CDA* nucleotide and protein sequences for *CDA* genes from *T. castaneum*, *D. melanogaster*, *N. lugens*, *L. migratoria*, *Anopheles gambiae* (Dipteran: Culicidae) were used as a reference (Supp Table S2 [online only]) to assemble transcriptome and genome databases using Geneious R9 software (Kearse *et al.* 2012). Subsequently, each of the putative CDA-like sequences was identiﬁed used BLAST tool (https://blast.ncbi.nlm.nih.gov/Blast.cgi/).

Reverse-transcription PCR (RT–PCR) was used to validate *CDA* gene sequences screened from the genome and transcriptome database of *S*. *furcifera*. Primers were designed using Primer Premier 6.0 (Premier Biosfot, CA, USA; Supp Table S1 [online only]). RT–PCR was performed using Taq DNA polymerase (Catalog numbers: B600001, Sangon Biotech, Shanghai, China), with reaction program as follows: initial denaturation at 94°C for 2 min, followed by 35 cycles of denaturation at 94°C for 30 s, annealing at 55–60°C for 30 s, and elongation at 72°C for 1–2 min, with extension at 72°C for 10 min. PCR product purified were using a DiaSpim Column DNA Gel Extraction Kit (Catalog numbers: B110092, Sangon Biotech, Shanghai, China), and each purified DNA product was sequenced in both directions by Sangon Biotech (Shanghai, China).

Full-length cDNA sequences of *S*. *furcifera CDA* genes were cloned by rapid amplification of cDNA ends (RACE) using a SMARTer RACE cDNA Amplification Kit (Clontech, Mountain View, CA, USA) following the manufacturer’s instructions. Gene-specific primers (GSPs) for ampliﬁcation of 5’RACE and 3’RACE were designed based on the known fragments of CDA genes, the primers listed in Supp Table S1 (online only). The first PCR was performed using the universal primer mix provided (UPM) and the GSP. PCR conditions were as follows: followed by 25 cycles at 94°C for 30 s, annealing at 60–70°C for 30 s, and elongation at 72°C for 3 min. Then, the first PCR products were diluted 50–100 times for use as a cDNA template for the second PCR, which was performed using the common short primer and the GSP. Conditions for the second PCR were the same as those for the first PCR. PCR products were purified using a DiaSpim Column DNA Gel Extraction Kit, and each purified product was ligated into the pMDTM^18^-T Vector (Takara, Japan) and transformed into DH5α competent cells (Sangon Biotech, Shanghai, China). Positive clones were selected for Sangon Biotech sequencing.

### Sequence Analysis and Phylogenetic Tree Construction

ORF Finder (https://www.ncbi.nlm.nih.gov/orffinder/) was used to accurately identify open reading frames and protein-coding amino acids for the *CDA* genes. The cDNA sequence and deduced amino acid sequences of *SfCDAs* were analyzed using DNAMAN 6.0 (LynnonBiosoft, Quebec, Canada). The molecular weight (MW) and isoelectric point (PI) of the protein were predicted using ExPASy (https://web.expasy.org). Domain structures were predicted using SMART (http://smart.embl-heidelberg.de/). Alignments of deduced amino acid sequences for the *CDAs* were assessed using the BoxShade service (https://www.expasy.org/resources/boxshade). A phylogenetic tree of eight insects protein CDA sequence was constructed using MEGA 6.0 software (Tamura *et al.*, 2013), adopting the maximum likelihood (ML), neighbor-joining (NJ), and maximum parsimony (MP) algorithm with 1000-fold bootstrap resampling.

### Developmental and Tissue-Specific Expression Analysis of Four *SfCDA*

To perform developmental and tissue-specific expression analysis of *SfCDA*, samples at 21 different developmental stages (including eggs, first- to fifth-instar nymphs, 1- to 4-d old male and female adults) were collected. Six different tissues including integument, gut, fat body, head, leg dissected from the fifth-instar nymphs, and integument, gut, fat body, head, leg, wing, ovary, and testis were collected from third to fourth day male or female adult. Total RNA was isolated using an HP Total RNA kit (Omega Bio-Tek, Norcross, GA, USA) according to the manufacturer’s instructions. The concentration and quantity of total RNA were assayed using a spectrophotometer (NanoDrop 2000, Thermo, USA). One microgram of total RNA was used to synthesize ﬁrst-strand complementary DNA (cDNA) by using PrimeScript RT reagent kit with gDNA Eraser (TaKaRa, Japan) according to the manufacturer’s protocols. The expression profiles of the genes for the four *SfCDAs* were determined using quantitative real-time PCR (RT–qPCR), The following conditions were applied: initial denaturation for 10 min at 95°C followed by 40 cycles, at 95°C for 30 s, and 60°C for 30 s with CFX96 RT–qPCR system (Bio-Rad, Hercules, CA, USA) using FastStart Essential DNA Green Master Mix (Roche, Indianapolis, IN, USA). Melting curve stage was tested for 0.5 s at 65°C to 95°C. The ribosomal protein L9 (RPL9, Accession number: KM885285) was used as an internal reference gene. All RT–qPCR experiments analyses were repeated in triplicate, and 2^−ΔΔCt^ method was used to determine the relative quantification (Livak and Schmittgen., 2001). The primers for RT–qPCR are shown in Supp Table S3 (online only). At the same time, we used semi-quantitative RT–PCR to detect the four *SfCDAs* expressions in different tissues. The RNA extraction and ﬁrst-stand cDNA synthesis method were followed as described above.

### RNA Interference

Specific primers for each *CDA* gene were designed using Primer Premier 6.0, with each primer containing a T7 polymerase promoter sequence. The dsRNA primers are shown in Supp Table S3 (online only). The dsRNAs were synthesized using a Transcript Aid T7 High Yield Transcription Kit (Thermo, Waltham, MA, USA), following the manufacturer’s protocol. The integrity of the dsRNA was verified on a 1.2% agarose gel, and dsRNA concentrations were determined using a NanoDrop 2000 spectrophotometer. The dsRNA for GFP (Accession number: CAA58789) was used as a nonspecific negative control gene.

To study the biological role of *CDA* genes in the molting process of *S*. *furcifera*, we used first day fifth-instar nymphs for micro-injection experiments. Each insect was injected with 1000 ng/μl gene-specific dsRNA using a microinjector (IM-31 Injector; Narishige, Tokyo, Japan). There were 120 insects for three biological replicates. Each biological replicate included forty insects. Post-injection insect transferred onto at glass tubes in fresh rice seedlings and mortality rates were recorded and phenotypes were observed on a daily basis. To determine silencing efficiency, 10 nymphs were randomly chosen for RNA extraction at 48 and 72 h after dsRNA injection. mRNA-level detection of target genes was performed using RT–qPCR of extracts.

### Expression of Chitin Biosynthesis Genes After RNAi of *SfCDA1, SfCDA2, SfCDA3,* and *SfCDA4*

To determine the effects of RNAi on the transcript expression of genes involved in chitin biosynthesis-chitin synthase 1 (*SfCHS1*) (Wang *et al.*, 2019) and trehalase (*SfTRE*), after 72 h injected ds*SfCDA1*, ds*SfCDA2*, ds*SfCDA3*, and ds*SfCDA4*, nymphs were collected to extract RNA and synthesize cDNA. The expressions levels of these genes were analyzed by RT–qPCR as described above. Primer are listed in Supp Table S4 (online only).

### Statistical Analysis

Statistical analyses were performed using SPSS 21.0 (Chicago, IL, USA), and data are presented as standard errors of the mean. One-way ANOVA followed by a Tukey test was used to analyze relative expression levels at different development stages and different tissues. Student’s *t*-test was used to compare the dsRNA treatment and control groups. Kaplan–Meier (log-rank: Mantel–Cox) tests used GraphPad Prism 8.0 to analyze survival rates.

## Results

### Identification and Analysis of *SfCDA* Sequence

In total, we identified four CDA genes (*SfCDA1*, *SfCDA2*, *SfCDA3*, and *SfCDA4*) from the *S*. *furcifera* genome and transcriptome database. Each CDA gene fragment was conﬁrmed by RT–PCR and sequencing, and used the RACE method to amplify four CDA gene full-length sequences. The gene names, cDNA length, encoding protein, theoretical isoelectric point, molecular weight, and accession number for the four *S*. *furcifera CDA* genes are shown in Table 1.

**Table 1.** Characteristics of chitin deacetylase genes in *S*. *furcifera*

Group	Gene	cDNA length	Coding region	5’UTR	3’UTR	Aa	pl	Mw (KDa)	GenBank ID
Group I	*SfCDA1*	2745	165–1811	164	934	548	5.4	56.297	MN508364
	*SfCDA2*	2506	160–1803	159	703	547	5.1	62.326	MN482711
Group Ⅲ	*SfCDA3*	2172	117–1601	116	571	494	5.5	56.454	MN482712
Group Ⅳ	*SfCDA4*	3888	367–2799	366	1089	810	6.2	91.806	MN482713

**Fig. 1.**
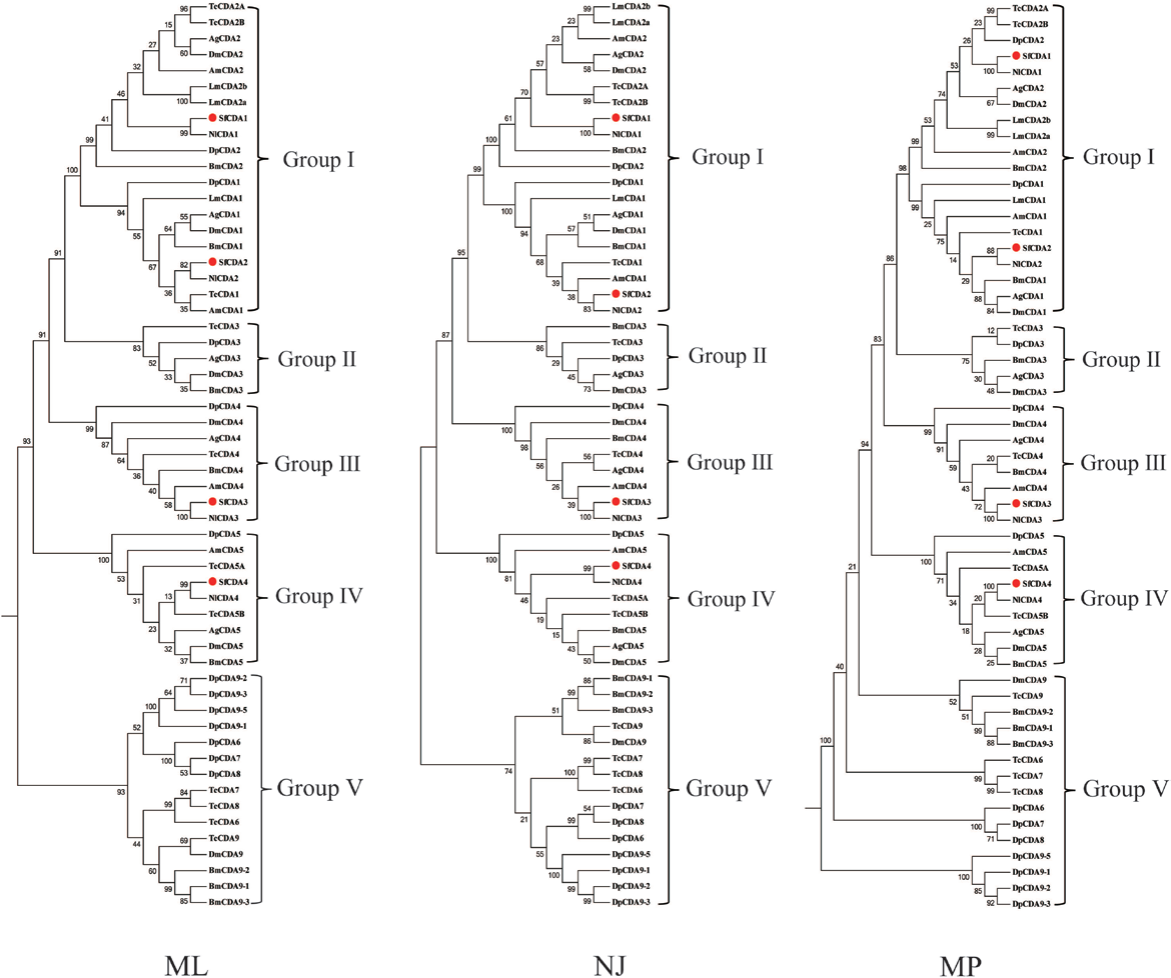
Phylogenetic analyses of genes for chitin deacetylases (CDAs) from *Bombyx mori* (Bm), *Drosophila melanogaster* (Dm), *Tribolium castaneum* (Tc), *Anopheles gambiae* (Ag), *Locusta migratoria* (Lm), *Nilaparvata lugens* (Nl), *Daphnia pulex* (Dp), and *Sogatella furcifera* (Sf). The genes for CDAs were classified into five groups (I–V). The GenBank accession numbers for all of the insect protein sequences for CDAs used are listed in Supp Table S2 (online only).

Three phylogenetic tree analysis was performed to investigate the evolutionary relationship of CDAs from *S*. *furcifera* and other organisms (Fig. 1). The insect *CDA* genes were categorized into five groups (I–V). *SfCDA1* and *SfCDA2* were clustered into group I, *SfCDA3* belonged to group III, and *SfCDA4* was placed in group IV. No *S*. *furcifera CDA*s were classified into groups II and V.

### Domain Architecture and Catalytic Domains of CDA Protein Sequences

**Fig. 2.**
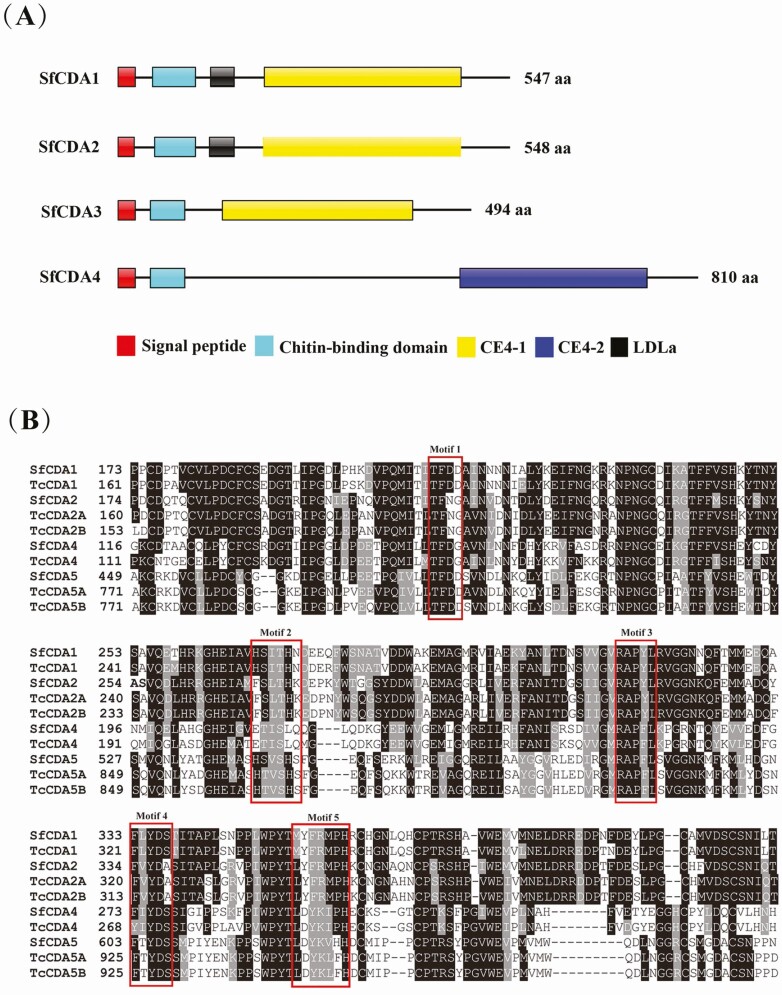
Deduced domain architectures and catalytic domain of four CDAs from *S. furcifera*. (A) The deduced amino acid sequences were used to predict the domain architectures of four CDAs in *S. furcifera* using SMART software. (B) Multiple sequence alignments of catalytic domains of the deduced genes for CDAs in *T. castaneum* and *S. furcifera*. The red boxes marked 1–5 indicate that the catalytic domain motif is TFDD, H[S/T]xxHP, RxP[Y/F], FxYD[S/A], or Lxxxx[P/F]H, respectively.

Domain architecture was predicted from deduced protein sequences of the four *S. furcifera CDA* genes using the online SMART software (Fig. 2A). All four CDA genes contained one CE4 polysaccharide deacetylase, a CBD (pfam01607), and a signal peptide. Specifically, *SfCDA1, SfCDA2*, and *SfCDA3* had a CE4-like 1 domain (cd10974), whereas *SfCDA4* had a CE4-like 2 domain (cd10975); *SfCDA1 and SfCDA2* had an LDLa (SM00192), whereas *SfCDA3* and *SfCDA*4 lacked an LDLa.

We identified five signature motifs (motifs 1–5) in *SfCDA1, SfCDA2, SfCDA3*, and *SfCDA4*: TFDD, H[S/T]xxHP, RxP[Y/F], FxYD[S/A], and Lxxxx[P/F]H, where x denotes a nonspecific amino acid (Fig. 2B). These results are consistent with those reported in *T. castaneum* (Dixit *et al.*, 2008).

### Developmental and Tissue Expression Patterns of *SfCDAs*

**Fig. 3.**
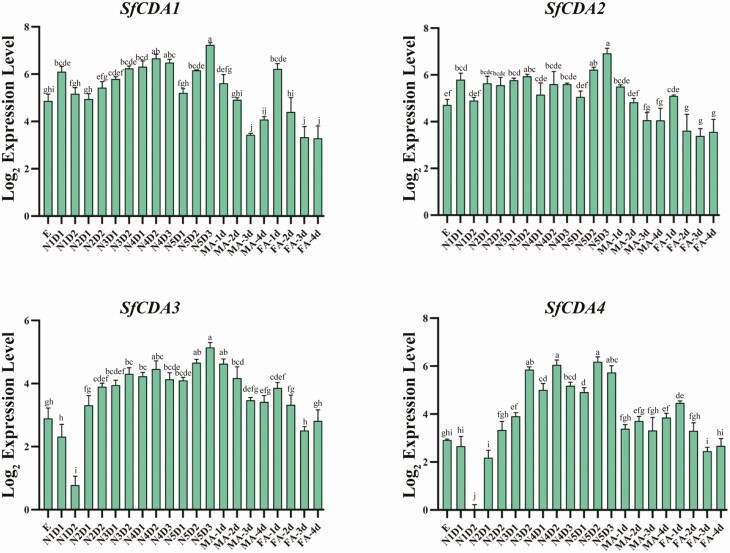
Developmental expression analyses of four chitin deacetylase genes in *S. furcifera*. E, Egg; N1D1–N1D2, 1- to 2-d-old first-instar nymphs; N2D1–N2D2, 1- to 2-d-old second-instar nymphs; N3D1–N3D2: 1- to 2-d-old third-instar nymphs; N4D1–N4D3, 1- to 3-d-old fourth-instar nymph; N5D1–N5D3, 1- to 3-d-old fifth-instar nymph; MA-1-4d: 1- to 4-d-old male adults; and FA-1-4d: 1- to 4-d-old female adults. Data are presented as standard error of the mean of three biological replications, analyzed. The lowercase letters above the bars indicate significant differences (*P* < 0.05).

The developmental expression patterns of four *SfCDAs* were determined using RT–qPCR (Fig. 3). *SfCDA1* and *SfCDA2* had similar expression patterns, with transcript expressed at all nymph stages of development at a stable level. *SfCDA3* and *SfCDA4* were expressed at a low level from egg to the second-instar nymphs, but *SfCDA3* and *SfCDA4* mRNA expressions were higher from third-instar to fifth-instar nymphs. Subsequently, *SfCDA1, SfCDA2, SfCDA3*, and *SfCDA4* were expressed at gradually decreasing levels in the male and female adult stages.

**Fig. 4.**
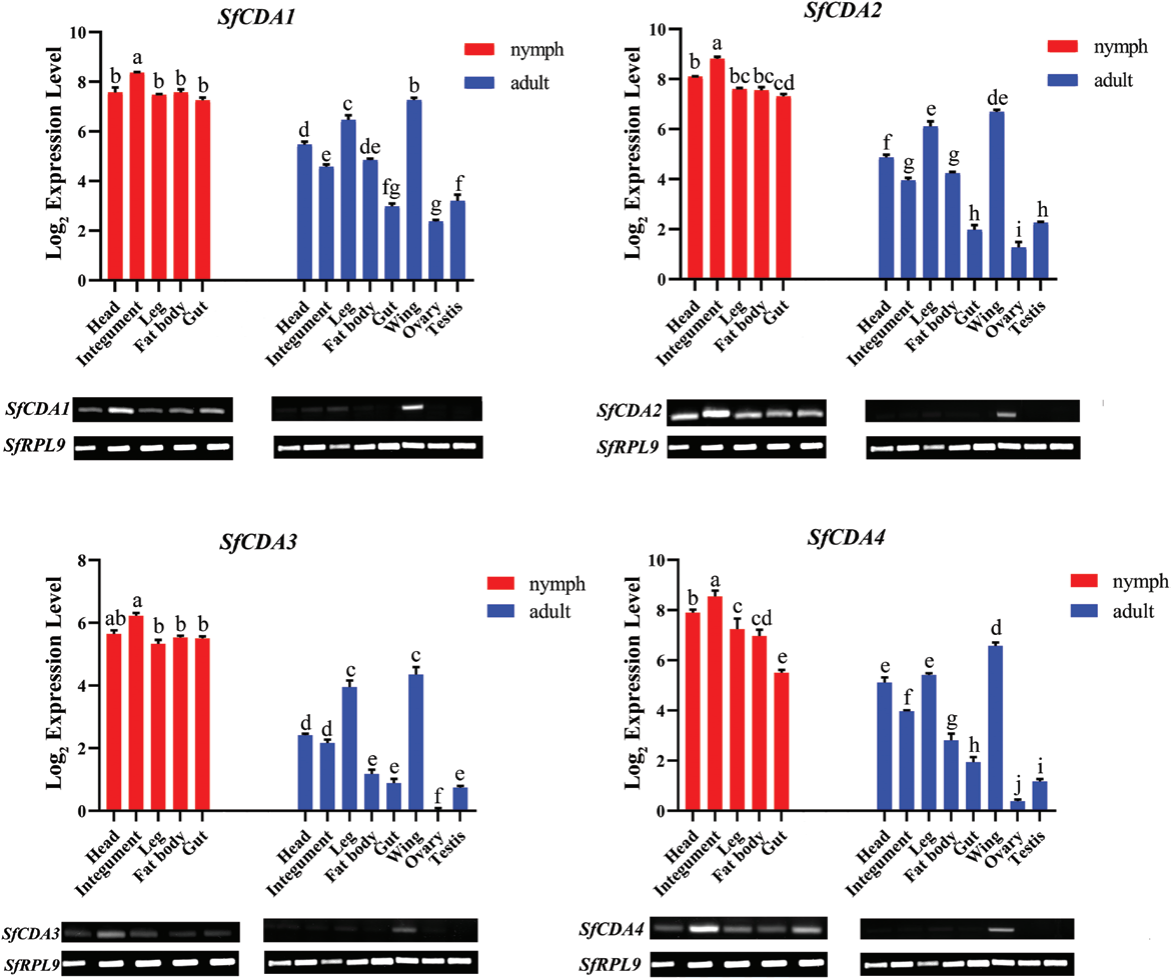
Tissue-specific expression analyses of four chitin deacetylases genes in *S. furcifera*. Data are presented as standard error of the mean of three biological replications, analyzed with one-way analysis of variance (ANOVA), and followed by Tukey’s multiple comparison test. The lowercase letters above the bars indicate significant differences (P < 0.05).

The expression patterns of the four CDA genes were determined by RT–qPCR in different tissues (Fig. 4). *SfCDAs* were expressed at highly varied levels among nymph and adult tissues. In fifth-instar nymph tissues, the *SfCDA1, SfCDA2, SfCDA3*, and *SfCDA4* were all highly expressed in the integument, head, fat body, leg, and gut (Fig. 4). In adults tissues, all four CDA genes were mainly expressed in the wing, leg, and head, while they were expressed at extremely low levels in the other adult tissues (Fig. 4).

### Functional Analyses of CDA Genes Through RNAi

**Fig. 5.**
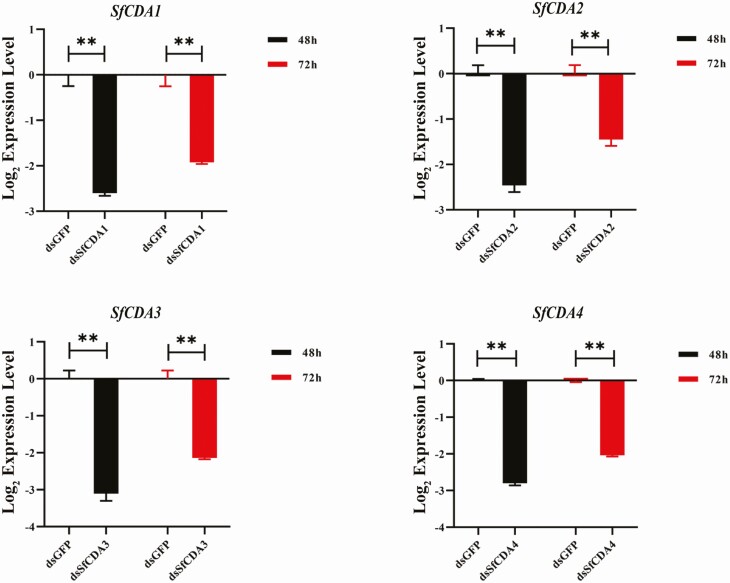
RT–qPCR analyses of silencing efficiency of the injection of double-stranded RNA (dsRNA) for 48 and 72 h. The data are presented as standard error of the mean of three biological replications. At 48 and 72 h after dsRNA injection, the expression was significantly decreased compared to controls (*t*-test, **P < 0.01).

To explore the biological function of the four CDA genes on *S. furcifera* molting, we performed functional analyses of CDAs by RNAi. The dsRNAs for *SfCDA1*, *SfCDA2*, *SfCDA3*, and *SfCDA4* were injected into first day fifth-instar nymphs, and dsGFP as a control. RT–qPCR results showed that transcriptional was significantly inhibited at 48 and 72 h after dsRNA injection (Fig. 5).

**Fig. 6.**
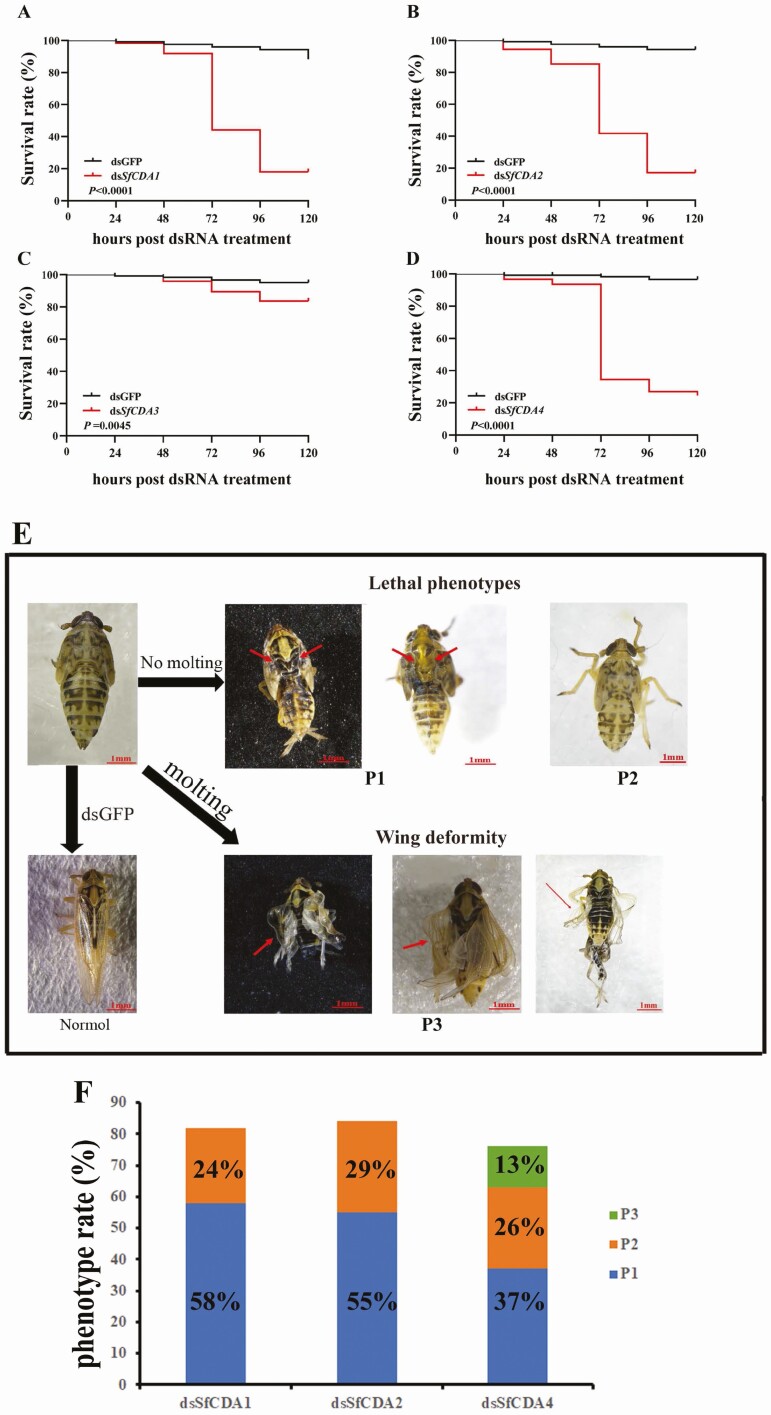
Effects of *SfCDA1*, *SfCDA2*, *SfCDA3*, and *SfCDA4* RNAi on the nymph–adult transition in *S. furcifera*. (A–D) Survival rate after ds*SfCDA1*, ds*SfCDA2*, ds*SfCDA3*, and ds*SfCDA4* or dsGFP injection. Survival rates were estimated using the Kaplan–Meier method. (E) For injection of dsRNAs for *SfCDA1*, *SfCDA2*, and *SfCDA4*, two lethal phenotypes were observed. P1: The old cuticle split open, but the nymph failed to shed the old cuticle and died before molting (red arrows). P2: The old cuticles did not split and the nymph failed to reach the molting stage, dying before eclosion. P3: Although the nymph could emerge as an adult, the individual adult had wing deformity (red arrows).

At 96 h following inhibition of *SfCDA1* and *SfCDA2*, the survival rate of nymphs was 18% and 16%, respectively (Fig. 6A and B). Two lethal phenotypes were observed after the injection of dsRNAs for *SfCDA1* and *SfCDA2*. In Phenotype 1, nymph old cuticles of the head and thorax are only slightly splitted open and eventually lead to death. In Phenotype 2, old nymph cuticles did not split from the head and thorax and died before molting (Fig. 6E). Injection of ds*SfCDA1* resulted in 82% of nymphs lethal phenotypes. Of 82% of nymph lethal phenotypes, the proportion of P1 was 58% and in P2, 24%, respectively (Fig. 6F). Injection of ds*SfCDA2* resulted in 84% of nymphs lethal phenotypes. Of 84% of nymph lethal phenotypes, the proportion of P1 and P2 was 55% and 29%, respectively (Fig. 6F).

At 96 h after injection of dsRNAs for *SfCDA4*, the normal emerged rate of nymphs was 23.4% (Fig. 6D). Three phenotypes were observed after injection of ds*SfCDA4*. P1 and P2 were similar to those caused by ds*SfCDA1* and ds*SfCDA2* (Fig. 6E). In addition, another abnormal phenotype was observed in adults. In P3, although nymphs could morph into adults, but had curled wings and could not spread them normally. (Fig. 6E). Injection of ds*SfCDA4* resulted in 63% of nymphs lethal phenotypes and 13% with adult curled wings. Of 63% of nymphs lethal phenotypes, the proportion of P1 and P2 were 37% and 26% (Fig. 6F).

Nymphs injected with dsRNAs for *SfCDA3* could successfully molt and develop into adults, and no significant phenotypic changes were observed. Their survival rate was 84% at 96 h following injection of ds*SfCDA3* (Fig. 6C).

### Knockdown of *SfCDA1, SfCDA2, SfCDA3*, and *SfCDA4* Affects the Expression of Chitin Bioynthesis Genes

**Fig. 7.**
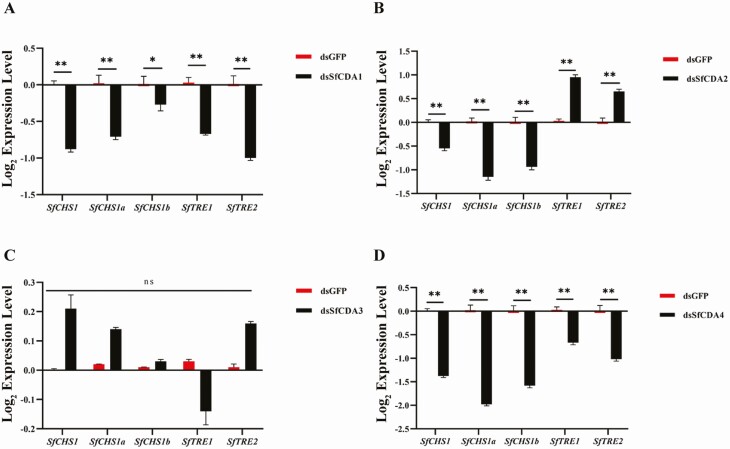
Relative expression levels for chitin synthesis pathway after RNAi. (A–D) The relative expression levels of chitin synthesis (*SfCHS1, SfCHS1a, SfCHS1b, SfTRE1*, and *SfTRE2*) in *S. furcifera* at 72 h after injection of dsRNA of *SfCDA1, SfCDA2, SfCDA3,* and *SfCDA4* (*t*-test; *P < 0.05, **P < 0.01).

We used RT–qPCR to detect the expression of chitin synthesis pathway genes (*SfCHS1, SfCHS1a, SfCHS1b, SfTRE1*, and *SfTRE2*) after RNAi of *SfCDAs* genes for 72 h in first day fifth-instar nymphs. The transcript levels of *SfCHS1*, *SfCHS1a*, and *SfCHS1b* were down-regulated and significantly treated with ds*SfCDA1*, ds*SfCDA2,* and ds*SfCDA4* compared with the control (Fig. 7A, B, and D). In addition, expression of *SfTRE1* and *SfTRE2* was significantly decreased compared with controls when *SfCDA1* and *SfCDA4* were silenced, while expression of *SfTRE1* and *SfTRE2* was increased significantly compared with controls after *SfCDA2* was silenced (Fig. 7A, B, and D). This suggests that *SfCDA1, SfCDA2,* and *SfCDA4* are involved in the regulation of the chitin biosynthesis genes during the nymph–adult transition. However, there was no significant change in the expression of the five chitin biosynthesis path genes after injection of *SfCDA3* RNAi, relative to levels in controls (Fig. 7C).

## Discussion

CDA is a key enzyme involved in chitin metabolism. Numerous *CDA* family genes have been identified in many insects species. There are four *CDA* genes in *N*. *lugens* (Xi *et al.* 2014), six in *D*. *melanogaster* (Dixit *et al.* 2008), nine in *T*. *castaneum* (Arakane *et al.* 2009), nine in *Manduca sexta* (Lepidoptera: Sphingidae) (Tetreau *et al.* 2015), and eight in *B. mori* (Zhang *et al.* 2019). In this study, we identified four full-length CDAs genes in *S. furcifera* by cloning and sequencing. Interestingly, we found no *CDA*s genes of *S*. *furcifera* belonging to groups II or V. This is in line with results in hemimetabolous insects in Hemiptera and Anapleura (Xi *et al.* 2014). Spatiotemporal expression analyses showed that *SfCDA1, SfCDA2, SfCDA3*, and *SfCDA4* upregulated expression during the molting period and mainly expressed in the integument, suggesting that these genes may be involved in molting development.

### Insect Group I CDAs

Group I contains genes encoding the proteins *CDA1* and *CDA2* in insects. In this study, *SfCDA1* and *SfCDA2* were expressed at all nymph developmental stages and in the integument, head, fat body, leg, and gut. Silencing of *SfCDA1* and *SfCDA2* caused high mortality or phenotypic defects (Fig. 6E). Similar results have been documented in hemimetabolous insects, such as with *CDA1* and *CDA2* in *N*. *lugens* (Xi *et al.* 2014), *Oxya chinensis* (Orthoptera: Acridoidea) (Ding *et al.* 2014; Yu *et al.* 2014), and *L*. *migratoria* (Yu *et al.* 2016; Yu *et al.* 2018). These findings further support the conclusion that *CDA1* and *CDA2* are indispensable during molting. In previous research, it was shown that group I CDAs in some insects have redundant functions. For example, in *D*. *melanogaster*, both serp (*CDA1*) and verm (*CDA2*) lead to elongated and tortuous tracheal tubes (Luschnig *et al.* 2006, Wang *et al.* 2006). Interestingly, Gal4/UAS expression system was used to tissue-speciﬁc RNA interference with serp and verm expression, demonstrating that serp and verm play distinct roles in wing development of *D. melanogaster* (Zhang *et al.* 2019). In the current study, *SfCDA1* and *SfCDA2* were both expressed adult wing, the silencing of *SfCDA1* and *SfCDA2* in the fifth-instar nymphs of *S*. *furcifera* resulted in 82% and 84% nymph mortality, but no adult wing deformity has been observed, respectively. We did not know whether application of Gal4/UAS expression system treatment with *SfCDA1* and *SfCDA2* has a similar eﬀect on *S*. *furcifera*, which remains unclear and is worth further researching.

### Insect Group III CDAs

Different insects show different expressions levels of group III *CDAs* at different developmental stages and in different tissues. In *M*. *sexta*, *MsCDA4* is expressed in all developmental stages and in the integument (Tetreau *et al.* 2015). The expression of *CmCDA4* in the head of *Cnaphalocrocis medinalis* (Lepidoptera: Pyralidae) and in the adult stage is significantly higher than that in the larval stage (Yu *et al.* 2016). However, while *TcCDA4* is highly expressed in the epidermis in both the larval and adult stages of *T*. *castaneum*, no obvious phenotypic changes are observed from silencing *TcCDA4* (Arakane *et al.*, 2009). Similarly, *L*. *migratoria CDA4* is highly expressed in the integument, but no visible abnormal phenotypic changes were observed after the injection of *dsLmCDA4*, and no effect is seen on content or organization of the chitin (Yu *et al.* 2017). In our study, *SfCDA3* was highly expressed in five nymph tissues (integument, head, fat body, leg, and gut) and were periodically expressed during the nymph molting stage, but knockdown of *SfCDA3* had no obvious effect on the development of *S*. *furcifera*. These data demonstrate that Group III CDA is not involved in molting development. Moreover, one *DcCDA3* from *Diaphorina citri* (Hemiptera: Chermidae) is highly expressed in the integument and third-instar nymph stage, but silencing of *DcCDA3* does not lead to any observable phenotypic change. Interestingly, injection of *Escherichia coli* and *Staphylococcus aureus* induces a significantly changed level of expression of *DcCDA3* in the midgut, suggesting that *CDA3* may be involved in the immune response of *D. citri* (Yu *et al.* 2020). Whether the *CDA3* of *S*. *furcifera* also has similar roles remains unknown and is worth further investigation.

### Insect Group IV CDAs

In *N*. *lugens*, RNAi silencing of *NlCDA4* causes arrested development in nymph–nymph transition and more than 95% mortality (Xi *et al.* 2014). In this study, we found that silencing of *SfCDA4* resulted in a lethal phenotype in 63% of nymphs and in 13% of abnormal wing adults. Similarity, among the chitin-degrading enzymes, *Cht7* in *S*. *furcifera* and *Plutella xylostella (Coleoptera: Plutellidae), β-N-acetylglucosaminidase 2* (*NAG2*) in *Lasioderma serricorne* (Coleoptera: Anobiidae) lead to wing development deformity (Chen *et al.* 2017, Zhu *et al.* 2019, Yang *et al.* 2019), indicating a chitin deposition critical role in adult wing development. By contrast, in *T*. *castaneum*, no phenotypic abnormality is observed after injection of ds*TcCDA5* (Arakane *et al.*, 2009). Likewise, in *L*. *migratoria*, silencing of *LmCDA5* shows no effect on locust development or cuticle structure (Yu *et al.* 2017), suggesting that the same *CDA* group gene may have different functions in different insects.

### Effects of *SfCDA1, SfCDA2, SfCDA3,* and *SfCDA4* RNAi on Transcripts of Chitin Biosynthesis

Our data indicate that *SfCDA1*, *SfCDA2*, and *SfCDA4* play a vital role in nymph–adult transition (Fig. 6E), but *SfCDA3* has no effect. The following question remained: Is there an influence on the transcript level of chitin synthesis after knockdown of the four *SfCDAs*? We found that injection of ds*SfCDA1*, ds*SfCDA2*, and ds*SfCDA4* reduced the level of transcript of *SfCHS1*, *SfCHS1a*, and *SfCHS1b*, and it increased or decreased the expression of *SfTRE1* and *SfTRE2* compared with controls, but RNAi suppression of *SfCDA3* expression did not affect expression of chitin biosynthesis genes (Fig. 7A–D). Similarly, in *Leptinotarsa decemlineata* (Coleopteran: Chrysomelidae), the mRNA levels of three transcripts (*LdChSAa*, *LdChSAb*, and *LdChSB*) are significantly lower, and those of two trehalose transcripts (*TRE1* and *TRE2*) are upregulated compared with controls, in *LdCDA1*- and *LdCDA2b*-depleted larvae (Wu *et al.* 2019a, Wu *et al.* 2019b). In *S*. *furcifera*, RNAi inhibited the expressions of *SfCht5*, *SfCht7*, *SfCht10*, and *SfIDGF2* and caused a change in the expression level of chitin synthesis *SfCHS1*, *SfTRE* (Yang *et al.* 2020). In *L. serricorne*, two chitin synthesis genes (*CHS1* and *TRE1*) were significantly downstream after knockdown of *LsNAG2* (Yang *et al.* 2019). Thus, these results showed that *CHS* and *TRE* expression levels were regulated by the *Cht*, *NAG*, and *CDA* genes.

In summary, we successfully cloned and characterized four full-length *CDA* genes from *S*. *furcifera*. These genes belong to three of the five distinct groups in the *CDA* family, but *S*. *furcifera* lacks orthologues in groups II and V found in hemimetabolous insects. *SfCDA1* and *SfCDA2* were expressed at all nymph developmental stages, and *SfCDA3* and *SfCDA4* were mainly expressed in the third-instar to fifth-instar nymph stages. Furthermore, *SfCDA1*, *SfCDA2*, *SfCDA3*, and *SfCDA4* were predominantly expressed in the integument and head, followed by the adult wing, whereas RNAi results showed that *SfCDA1*, *SfCDA2*, and *SfCDA4* are involved in the nymph–adult molting transition and these genes could regulate chitin biosynthesis expression levels.

## Supplementary Material

ieab051_suppl_Supplementary_Material
