# Supplementary material for: Identification and RNAi-Based Functional Analysis of Four Chitin Deacetylase Genes in Sogatella furcifera (Hemiptera: Delphacidae)
Source: J Insect Sci. 2021 Aug 1;21(4):9. doi: 10.1093/jisesa/ieab051 (PMC8325873; doi:10.1093/jisesa/ieab051)
Supplement: ieab051_suppl_Supplementary_Material [file ieab051_suppl_supplementary_material.pdf]

Table S1. Primers used for RT-PCR and RACE reaction.

PCR reactions	Primer name	Primer sequence（5'-3'）
RT-PCR	SfCDA1-F1	TGCTGCTCGCTCTCATTCT
	SfCDA1-R1	CGTTGATGGCACCGTTGAA
	SfCDA1-F2	ACGCTTGCTCTGTGGAGTC
	SfCDA1-R2	GAAGTGACGGTTGAAGTTGTGA
	SfCDA1-F3	CGCACAAGTGCAACGGTAA
	SfCDA1-R3	CACTGATCGGTCGCTCTGT
	SfCDA2-F1	TGCTGCTTCCGTCTGTCTG
	SfCDA2-R1	GCGATGTTGTTGTTGTTGATGG
	SfCDA2-F2	AGGTCTGTTCTGTAACGGAGAG
	SfCDA2-R2	CGAAGTTGTGGTTGAGGAAGTT
	SfCDA2-F3	ACCAGGAGTCACGCTGTATG
	SfCDA2-R3	GCTGCCTCGCTCCAGATAA
	SfCDA3-F1	GAGCAGGTAACGGCAACTATG
	SfCDA3-R1	AGGAACGGAGCACGCATT
	SfCDA3-F2	ACTGCGACTACAACATGATTCA
	SfCDA3-R2	GCTCGGTCATCCAGGTCAA
	SfCDA3-F3	GTCAGGCACTTGTCCTACTAA
	SfCDA3-R3	CGATTCTCCCATTCATTACTTC
	SfCDA4-F1	ACAGTCTGCTCGCTAGTGAT
	SfCDA4-R1	TGCTTCATCGTCGTAGAGGTT
	SfCDA4-F2	ACAGCAGCAGCAGCAACT
	SfCDA4-R2	CGTTGTAATCCTGTGCGTTGA
	SfCDA4-F3	TCCAGAATGACGACATCCAGTA
	SfCDA4-R3	CGCTGGTTTCTATCTTAGTTGG
5’RACE	SfCDA1-GSP	GATTACGCCAAGCTTCGTTGAATGTGATGGTGAT
	SfCDA2-GSP	GATTACGCCAAGCTTGAGCGATGTTGTTGTTGTT
	SfCDA3-GSP	GATTACGCCAAGCTTTCGCAGCCATTCGGATTA
	SfCDA4-GSP	GATTACGCCAAGCTTAACTCGTGGCTGTACATG
3’RACE	SfCDA1-GSP	GATTACGCCAAGCTTCGCTAACAACAAGAGAACT
	SfCDA2-GSP	GATTACGCCAAGCTTCGATCTCATCTCAATCAAACG
	SfCDA3-GSP	GATTACGCCAAGCTTGTGGAAGTGTGAGAAGAGG
	SfCDA4-GSP	GATTACGCCAAGCTTATTGTGTTCAGGCATTGAC

Table S2 Accession numbers of the insect CDA genes.

Accession number	Gene Name	Species	Order
XP_320597	*AgCDA1*	*Anopheles gambiae*	Diptera
XP_320596	*AgCDA2*	*Anopheles gambiae*	Diptera
XP_317336	*AgCDA3*	*Anopheles gambiae*	Diptera
XP_310753	*AgCDA4*	*Anopheles gambiae*	Diptera
XP_316929	*AgCDA5*	*Anopheles gambiae*	Diptera
NP_730444	*DmCDA1*	*Drosophila melanogaster*	Diptera
NP_001163469	*DmCDA2*	*Drosophila melanogaster*	Diptera
NP_609806	*DmCDA3*	*Drosophila melanogaster*	Diptera
NP_728468	*DmCDA4*	*Drosophila melanogaster*	Diptera
NP_001097044	*DmCDA5*	*Drosophila melanogaster*	Diptera
NP_611192	*DmCDA9*	*Drosophila melanogaster*	Diptera
NP_001095946	*TcCDA1*	*Tribolium castaneum*	Coleoptera
NP_001096047	*TcCDA2a*	*Tribolium castaneum*	Coleoptera
NP_001116303	*TcCDA2b*	*Tribolium castaneum*	Coleoptera
NP_001104011	*TcCDA3*	*Tribolium castaneum*	Coleoptera
NP_001103903	*TcCDA4*	*Tribolium castaneum*	Coleoptera
NP_001103739	*TcCDA5a*	*Tribolium castaneum*	Coleoptera
NP_001107799	*TcCDA5b*	*Tribolium castaneum*	Coleoptera
NP_001103905	*TcCDA6*	*Tribolium castaneum*	Coleoptera
NP_001104012	*TcCDA7*	*Tribolium castaneum*	Coleoptera
NP_001103906	*TcCDA8*	*Tribolium castaneum*	Coleoptera
NP_001103904	*TcCDA9*	*Tribolium castaneum*	Coleoptera
BGIBMGA006213	*BmCDA1*	*Bombyx mori*	Lepidoptera
BGIBMGA006214	*BmCDA2*	*Bombyx mori*	Lepidoptera
BGIBMGA008988	*BmCDA3*	*Bombyx mori*	Lepidoptera
BGIBMGA010573	*BmCDA4*	*Bombyx mori*	Lepidoptera
BGIBMGA002696	*BmCDA5*	*Bombyx mori*	Lepidoptera
BGIBMGA013756	*BmCDA9-1*	*Bombyx mori*	Lepidoptera
BGIBMGA013757	*BmCDA9-2*	*Bombyx mori*	Lepidoptera
BGIBMGA013758	*BmCDA9-3*	*Bombyx mori*	Lepidoptera
EFX70874	DpCDA1	*Daphnia pulex*	Crustacea

EFX70875	DpCDA2	*Daphnia pulex*	Crustacea
EFX84707	DpCDA3	*Daphnia pulex*	Crustacea
EFX69367	DpCDA4	*Daphnia pulex*	Crustacea
EFX77211	DpCDA5	*Daphnia pulex*	Crustacea
EFX80381	DpCDA6	*Daphnia pulex*	Crustacea
EFX88070	DpCDA7	*Daphnia pulex*	Crustacea
EFX88071	DpCDA8	*Daphnia pulex*	Crustacea
EFX68639	DpCDA9-1	*Daphnia pulex*	Crustacea
EFX68640	DpCDA9-2	*Daphnia pulex*	Crustacea
EFX61239	DpCDA9-3	*Daphnia pulex*	Crustacea
EFX62775	DpCDA9-4	*Daphnia pulex*	Crustacea
EFX61415	DpCDA9-5	*Daphnia pulex*	Crustacea
KR537803.1	*LmCDA1*	*Locusta migratoria*	Orthoptera
KR537804.1	*LmCDA2a*	*Locusta migratoria*	Orthoptera
KR537805.1	*LmCDA2*	*Locusta migratoria*	Orthoptera
ANA78277.1	*LmCDA4*	*Locusta migratoria*	Orthoptera
AJQ20732.1	*NlCDA1*	*Nilaparvata lugens*	Hemiptera
AJQ20733.1	*NlCDA2*	*Nilaparvata lugens*	Hemiptera
AJQ20734.1	*NlCDA3*	*Nilaparvata lugens*	Hemiptera
AJQ20735.1	*NlCDA4*	*Nilaparvata lugens*	Hemiptera
MN482711	*SfCDA1*	*Sogatella furcifera*	Hemiptera
MN508364	*SfCDA2*	*Sogatella furcifera*	Hemiptera
MN482712	*SfCDA3*	*Sogatella furcifera*	Hemiptera
MN482713	*SfCDA4*	*Sogatella furcifera*	Hemiptera

Table S3 Primers used for RT-qPCR and dsRNA synthesis.

PCR reactions	Primer name	Primer sequence（5'-3'）
**RT-qPCR**	SfCDA1-F	TCCATCACGCACAATGACGAAGAA
	SfCDA1-R	GAACTGGTTGTTGCCTCCGACTC
	SfCDA2-F	TCACATTCAACGGTGCCATCAAC
	SfCDA2-R	GCCATCTCAGCCAACCAATCATC
	SfCDA3-F	ACACAGCCGCATGTCAACTACC
	SfCDA3-R	ATCTCGCAGCCATTCGGATTACG
	SfCDA4-F	GCAGTGGTGGCACAACAGGT
	SfCDA4-R	GTTCTTCCTCGGCTGGCTGATG
	SfRPL9-F	GGGCGAGAAGTACATCCGTAGG
	SfRPL9-R	GCGGCTGATCGTGAGACATCTT
**dsRNA synthesis**	SfCDA1-F	TAATACGACTCACTATAGGGTGGAGATGGTGCCTGTATA
	SfCDA1-R	TAATACGACTCACTATAGGGAACTGTTCTTCGTCATTGTG
	SfCDA2-F	TAATACGACTCACTATAGGGTGATTGGTTGGCTGAGATG
	SfCDA2-R	TAATACGACTCACTATAGGGGCTGAAGTGACGGTTGAA
	SfCDA3-F	TAATACGACTCACTATAGGGGACCGATTCCAACAACAAG
	SfCDA3-R	TAATACGACTCACTATAGGGTTCTCGCATACCAAGCATT
	SfCDA4-F	TAATACGACTCACTATAGGGCTTCACCTACGATTCTTCTATG
	SfCDA4-R	TAATACGACTCACTATAGGGGTTGTTGAGCCTGTTGATG
	GFP-F	TAATACGACTCACTATAGGGGCCAACACTTGTCACTACTT
	GFP-R	TAATACGACTCACTATAGGGGGAGTATTTTGTTGATAATGGTCG

Table S4. Chitin synthesis pathway genes primers used for RT-qPCR and gene accession numbers.

Genes	accession numbers	Primer name	Primer sequence（5’- 3’）
SfTRE1	MG869613	SfTRE1-F	GACTTCTGCTATGTGATATGC
		SfTRE1-R	GCTGTCCACCATCTGAATA
SfTRE2	MG869614	SfTRE2-F	GTGGTTGGATGCTGTTACTA
		SfTRE2-R	GAGATGTTTGTCGGGTAGAA
SfCHS1a	KY350143	SfCHS1a-F	CTTCGGTGTTTGGTTTCTT
		SfCHS1a-R	TGGGTAACATCATCATAGGA
SfCHS1b	KY350144	SfCHS1b-F	GAGAAGGCGAGAATAGCA
		SfCHS1b-R	GCAGCAAGAACACGATTA
SfCHS1		SfCHS1-F	GATTGGTCATTGGCTTCAGA
		SfCHS1-R	GTAATGTCTTGCTTCGTCAG
